# Structural and Functional Brain Mapping Correlates of Impaired Eye Movement Control in Parkinsonian Syndromes: A Systems-Based Concept

**DOI:** 10.3389/fneur.2018.00319

**Published:** 2018-05-07

**Authors:** Martin Gorges, Hans-Peter Müller, Jan Kassubek

**Affiliations:** Department of Neurology, University of Ulm, Ulm, Germany

**Keywords:** magnetic resonance imaging, diffusion tensor imaging, “resting-state” functional magnetic resonance imaging, neurodegenerative movement disorder, video-oculography, Parkinson’s disease, progressive supranuclear palsy, multisystem atrophy

## Abstract

The investigation of the human oculomotor system by eye movement recordings provides an approach to behavior and its alterations in disease. The neurodegenerative process underlying parkinsonian syndromes, including Parkinson’s disease (PD), progressive supranuclear palsy (PSP), and multisystem atrophy (MSA) changes structural and functional brain organization, and thus affects eye movement control in a characteristic manner. Video-oculography has been established as a non-invasive recording device for eye movements, and systematic investigations of eye movement control in a clinical framework have emerged as a functional diagnostic tool in neurodegenerative parkinsonism. Disease-specific brain atrophy in parkinsonian syndromes has been reported for decades, these findings were refined by studies utilizing diffusion tensor imaging (DTI) and task-based/task-free functional MRI—both MRI techniques revealed disease-specific patterns of altered structural and functional brain organization. Here, characteristic disturbances of eye movement control in parkinsonian syndromes and their correlations with the structural and functional brain network alterations are reviewed. On this basis, we discuss the growing field of graph-based network analysis of the structural and functional connectome as a promising candidate for explaining abnormal phenotypes of eye movement control at the network level, both in health and in disease.

## Introduction

More than half a century ago, Carl F. List concluded in his essay that abnormal oculomotor function frequently gives valuable information of both the localization and the pathoanatomy of an underlying disease process (1). Although eye movements in the diseased brain have been extensively studied since then, it has been only recently that several multimodal studies support an increasingly coherent understanding of the structural and functional brain organization correlates. Characteristic disturbances of eye movement control accompany ongoing pathology (2) and include saccade disturbances, e.g., gaze palsy (3), saccadized smooth pursuit (4), or executive oculomotor dysfunctions, e.g., increased anti-saccade errors (5).

This narrative review links current experimental evidence of human behavior as observed from eye movement recordings in parkinsonian syndromes, including Parkinson’s disease (PD), progressive supranuclear palsy (PSP), and multisystem atrophy (MSA) to what is known from neuroimaging studies in structural and functional brain architecture. Moreover, we discuss how the growing field of graph theory-based investigations of the structural and functional connectome might provide a more elaborated approach to the principles of functional architecture underlying human behavior.

## The Oculomotor System and its Relation to Higher Cognitive Processes

**Figure 1:**
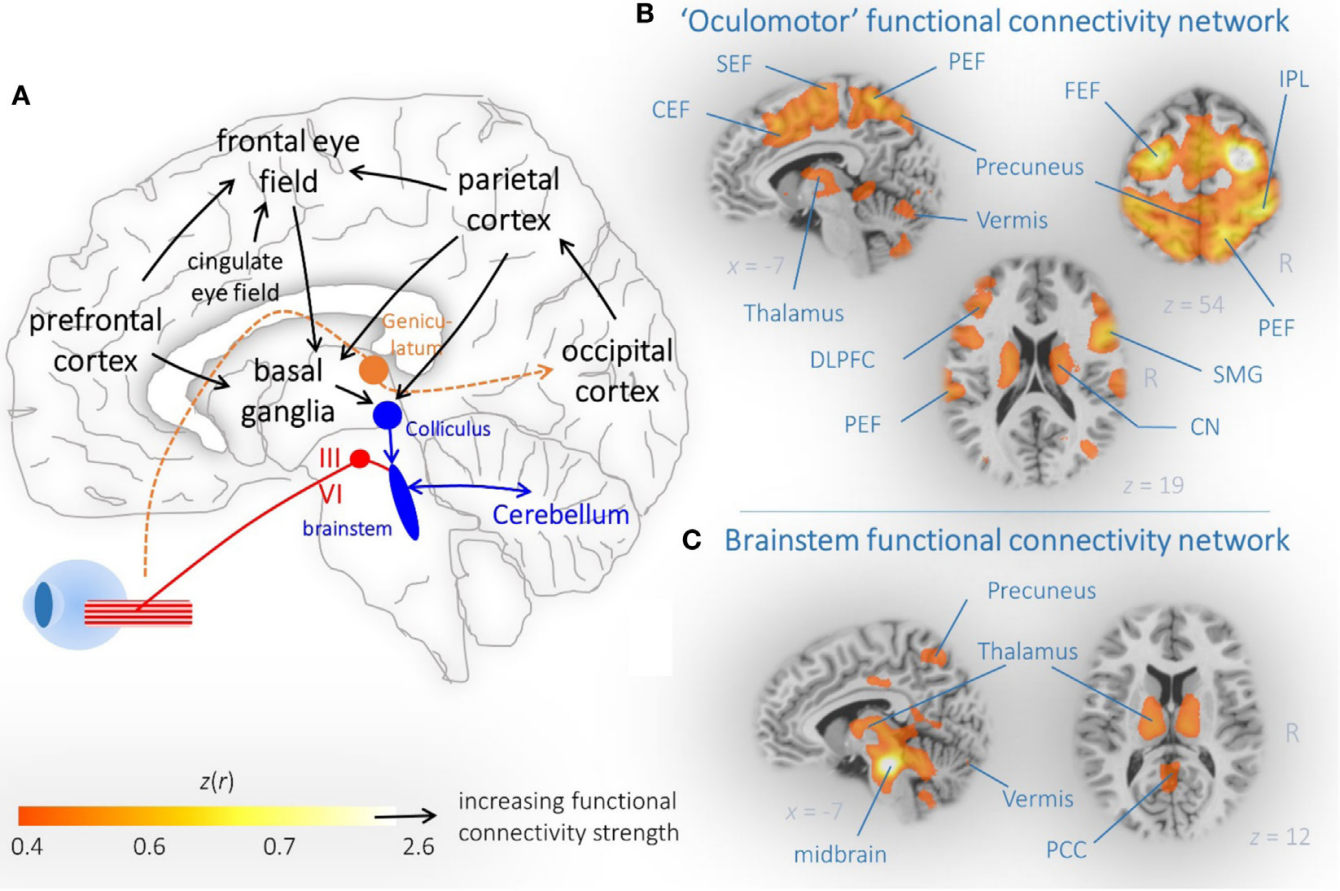
Brain networks associated with eye movement control. **(A)** Schematic illustration of cortical and subcortical brain regions that are critically involved in eye movement control. Arrows indicated the propagation of information. **(B)** Dorsal attention and **(C)** brainstem connectivity network reveal functionally coupled regions that are part of the oculomotor system. **(B,C)** “Resting-state” functional magnetic resonance imaging connectivity data from 12 healthy controls are shown as representative orthogonal brain section heat maps in the MNI stereotaxic space (19). The *z*(*r*) values indicate the strength of correlation that indicated functional coupling within the brain maps. Abbreviations: SEF, supplemental eye field; PEF, parietal eye field; CEF, cingulate eye field; IPL, interparietal sulcus; DLPFC, dorsolateral prefrontal cortex; CN, caudate nucleus; SMG, supramarginal gyrus; PCC, posterior cingulate cortex.

Human cognition is related to sensorimotor activation including oculomotion—a position that nowadays makes many researchers term eye movements as a window to complex forms of human behavior (6) and cognitive processes (7, 8). Subjects facing a choice between multiple stimuli tend to repeatedly look at them and more toward the option they are going to choose (8), presumably implementing a comparison process between different items (9). Brain structures and neural pathways which are involved in the control of eye movements have been reported in a multitude of studies (6, 10), as depicted in Figure 1. Brain mapping of eye movement control has been extensively studied in healthy human subjects including evidence from structural imaging (11), diffusion tensor imaging (DTI) (12), “task-evoked” (13), and “task-free” functional magnetic resonance imaging (fMRI) (14). These studies revealed that the control of eye movements involves multiple networks spanning the brainstem to the neocortex (15, 16). It is well known that parkinsonian syndromes present with progressive impairment of structural and functional brain networks (17), and it is a growing field of neuroimaging research how these brain alterations are linked with the respective oculomotor phenotype. In this context, getting subtle clues from abnormal eye movement control often requires standardized eye movement recordings with dedicated techniques, e.g., by means of video-oculography (18).

## Brain Mapping of Oculomotor Phenotypes in Neurodegenerative Parkinsonism

### Video-Oculographic Recordings of Eye Movements

Tracking eye movements with state-of-the-art video-based techniques is non-invasive and allows for precise and quantifiable measures of horizontal and vertical movements of the eye (20). Video-oculographic recordings have emerged as a tool in the diagnostic framework of vertigo (21) and especially of neurodegenerative movement disorders (22). Video-oculographic measurements are usually performed in a dedicated laboratory environment which is darkened, optically and acoustically shielded and provides a standardized experimental setup. The subject is comfortably placed in front of a screen with the head stabilized by a chin rest (18, 23). Infrared based light-weighted miniature cameras are mounted on a head band or helmet-like aperture and allow either binocular or monocular eye movement imaging (20). These recorded images are then automatically processed online or offline by a preconfigured computer that is usually an integral component of the eye tracking device (24). The computer provides orthogonal (i.e., horizontal and vertical) eye movement traces that can be analyzed in consideration of the presented stimuli. The stimulus design can incorporate smooth pursuit testing [e.g., by trapezoidal (25) or sinusoidal target motion (26)], reactive saccade testing (e.g., “jumping” target) (27), and executive function tests [e.g., anti-saccades (28)]. Smooth pursuit eye movement traces are analyzed for saccades that interrupt smooth pursuit. Recordings from reactive saccades, i.e., the performance of tracking a “jumping” target, are analyzed with respect to the primary saccades [with an eye amplitude of about 90–95% of the target amplitude (29)] and the reaction times, saccadic gain (i.e., saccade distance divided by target distance), and peak eye velocity. Executive functions testing such as anti-saccades address erroneous response (relative to number of elicited anti-saccades), i.e., pro-saccades toward the target are counted as an error because the subject is asked to immediately shift the gaze into the opposite direction with respect to the eccentric presentation of a visual target (28). Taken together, phenotyping of eye movement control allows for quantification of most useful parameters (such as peak eye velocity or smooth pursuit gain) which can potentially give clues to the clinician early in the course of a disease even when characteristic disease-defining symptoms are not overt (30).

### Characteristic but Non-Specific Eye Movement Patterns

**Figure 2:**
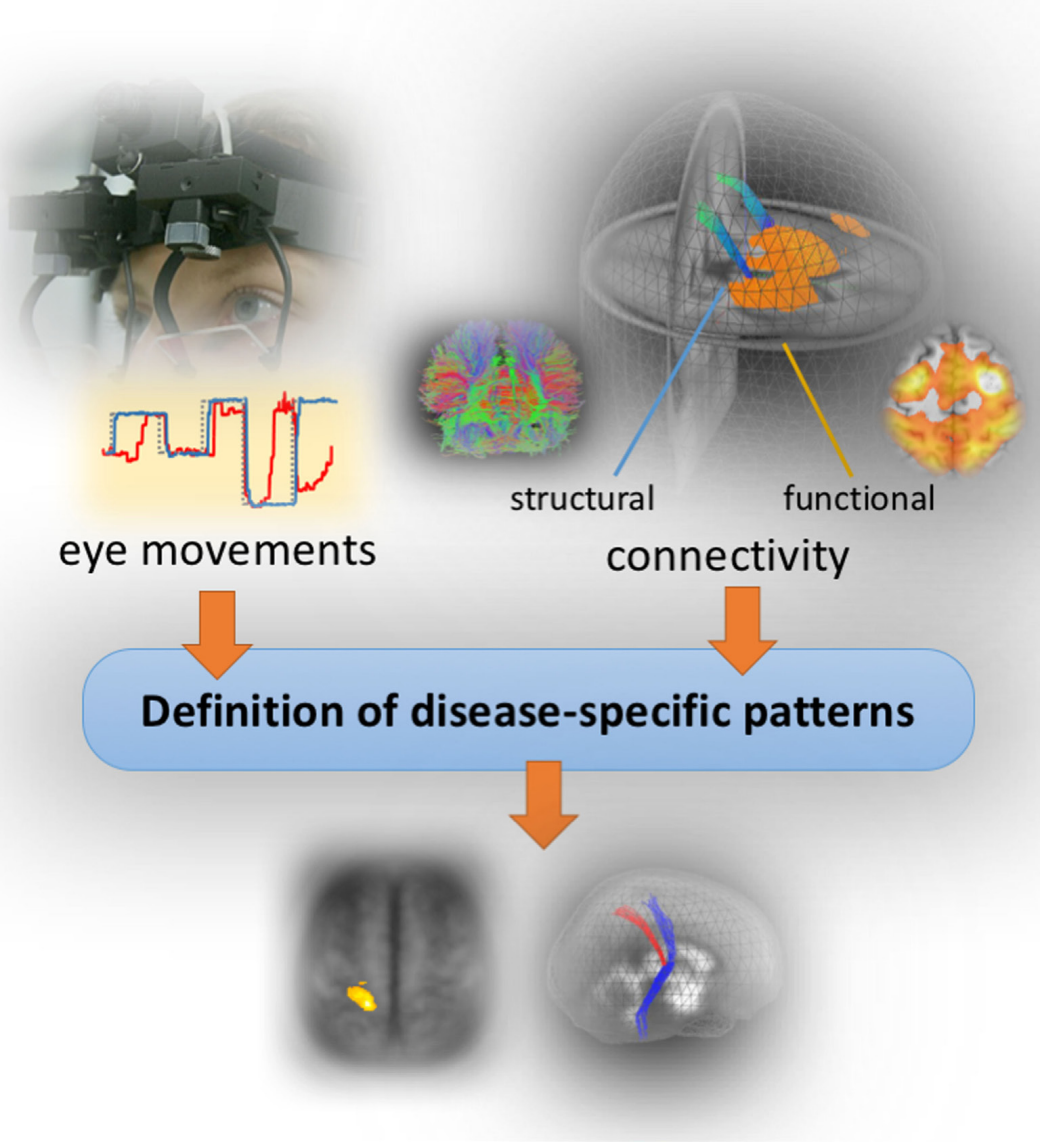
Concept of the bimodal study design. Structural and functional brain mapping of eye movement control by combining video-oculographically eye movements recordings (left upper panel) with structural and functional MR imaging data (right) in order to define disease-specific patterns.

Figure 2 illustrates a possible concept of mapping patterns of eye movement disturbances to brain structure and function. Oculomotor control examination is ideally performed at the time of MRI investigations in a dedicated oculomotor laboratory which allows a detailed investigation of eye movement control using state-of-the-art video-oculographically based tracing of eye movements in an acoustically shielded atmosphere (31) Deficits in eye movement control are generally present in parkinsonian syndromes (32). The patterns are characteristic for the phenotype but not disease-specific (22); under this prerequisite, quantification of eye movements is increasingly used as a functional investigation tool in the differential diagnostic framework (18).

### Brain Structural Correlates of Eye Movement Control

A multitude of widely distributed brain regions, including the brainstem (33), basal ganglia (34), and higher centers covering almost the entire neocortex (35, 36) exerts ultimate control over both voluntary and involuntary eye movements (Figure 1A). Using a bimodal or multimodal approach of video-oculography and neuroimaging, morphological alterations in association with deficits in oculomotor control can be addressed in order to see whether distinct brain regions are attributable to eye movement performance (11). Formerly, the voxel-based morphometry approach, a well-known technique to detect region-specific gray matter atrophy (37), was often used for voxel-based lesion symptom mapping (38). Absolute quantification by volumetric techniques such as atlas-based volumetry (ABV) of high-resolution three-dimensional MRI (39) provides a valuable alternative approach. ABV is a dedicated unbiased computer-based technique for absolute regional brain volume quantification at the individual level (39) that allows for fully automated classification of patients with parkinsonian syndromes (40). Using a bimodal analysis of ABV and video-oculographically recorded eye movements allows to identify the relationship between impaired eye movement control and regional brain atrophy in neurodegenerative parkinsonism (41). As a different methodological approach, DTI allows for the *in vivo* investigation of the brain’s microstructure within cerebral networks (42) and provides a more subtle measure on microstructural alterations as compared to morphometric or volumetric analysis (43). Disruption of microstructural tissue integrity has been reported in many studies in neurodegenerative Parkinson syndromes and may prove valuable in supporting the diagnosis of PD, PSP, and MSA (44). In association with eye movement control, DTI allows to determine axonal bundles that propagate information on eye movement control (45).

### Brain Functional Correlates of Eye Movement Control

Functional magnetic resonance imaging has enabled researchers to investigate functionally activated regions when performing a task as compared to a baseline (“rest”) condition (46). The fMRI signals during the performance of saccadic eye movement experiments exhibit a consistent spatial pattern of co-activated brain regions, including fronto-subcortical-parietal regions, thalamus, striatum, and intraparietal cortex (13). Smooth pursuit eye movement performance activated the common oculomotor network (47) including dorsal cortical eye fields and cerebellum (48). “Task-based” fMRI studies have supported the notion that cognitive functions and sensorimotor eye movements are closely interacting with each other and have helped to develop a better understanding of network-level brain abnormalities in neurodegenerative disorders (49). The “classic” “task-evoked” fMRI concept, i.e., simultaneous eye movement recordings and visual stimulus presentation in the MRI scanner, have limitations including the restriction of MRI-dedicated eye movement recording devices and the “noisy” and uncomfortable environment. Some concepts have emerged that to overcome these limitations of eye movement recordings in the scanner by running the fMRI scan afterward or before performing extensive eye movement assessment in a dedicated oculomotor lab (50, 51). The observed activation patterns in these studies revealed regions that are on the one hand part of the well-known oculomotor network and on the other hand regions that are known to be functionally disrupted in PD (50, 52). The activation patterns in association with saccades impairment in PSP demonstrated that not only in the brainstem, but also cortical neuronal networks contributed to impaired saccadic eye movements in PSP (51).

Functionally involved brain regions in eye movement control can be accurately captured by “task-free” or “resting-state” fMRI experiments, where subjects quietly “rest” in the scanner (53, 54). Resting-state (rs)-fMRI has gained substantial insights into the organization of intrinsic activity patterns of the human brain (54–56), after the discovery of temporally coherent patterns of ongoing low-frequency BOLD fluctuations under “resting” conditions (53). These patterns, i.e., intrinsic functional connectivity networks, remarkably resemble the maps of task-evoked coactive brain regions (57) and reveal a more general picture of the functional brain organization (58). Some substantial advances in understanding brain architecture have emerged from the observation of spontaneous “ongoing” brain activity as measured indirectly *via* the rs-fMRI signal while subjects lying quietly in the scanner (56). Understanding eye movement control on the basis of functionally interacting brain regions topologically organized as functional connectivity networks put forward the understanding of underlying pathology of impaired eye movement control and behavioral interpretations of these intrinsic connectivity networks (59).

## Brain Networks and Oculomotor Disturbances in Parkinsonian Syndromes

### Parkinson’s Disease

Parkinson’s disease is now recognized as an age-related multisystem disorder with cardinal motor symptoms that manifest years after the initial onset of pathogenesis—a process that is virtually self-promoting in a well predictable distribution pattern and not subject to remission (60, 61). A broad spectrum of oculomotor disturbances comprising impaired smooth pursuit, hypometric saccades, prolonged latencies, increased anti-saccade errors that accompany the cardinal motor symptoms (2, 62, 63). In particular, PD-associated oculomotor deficits (2, 64, 65) were shown to be predominantly attributable to executive impairment, because PD patients present substantial difficulties in suppressing unwanted gaze shifts by frequently moving their eyes away from the target in the absence of any distractor, but almost instantaneously correct these involuntary eye movements by re-foveating the target (63). This behavior is typically observed during visually guided reactive saccade performance and can be quantified as the rate of saccadic intrusions (63). Correlation analysis indicated a significant relationship between an increased rate of saccadic intrusions and overall cerebral brain atrophy but not with specific brain regions (41). This result at the structural level is supported by DTI-based investigations which were utilized in order to delineate the axonal organization of the brain at the microstructural level (42). In PD, however, the whole-brain-based analysis of diffusion patterns did not reveal significant correlations between eye movement parameters, e.g., between the rate of saccadic intrusions as a measure for executive control, and regional microstructural damage (41). The correlation analysis between brain structure and eye movement control in PD thus suggested that the executive dysfunctions are more likely attributed to a cortical network disorder (63, 66), rather than to regional brain atrophy or regional microstructural damage.

**Figure 3:**
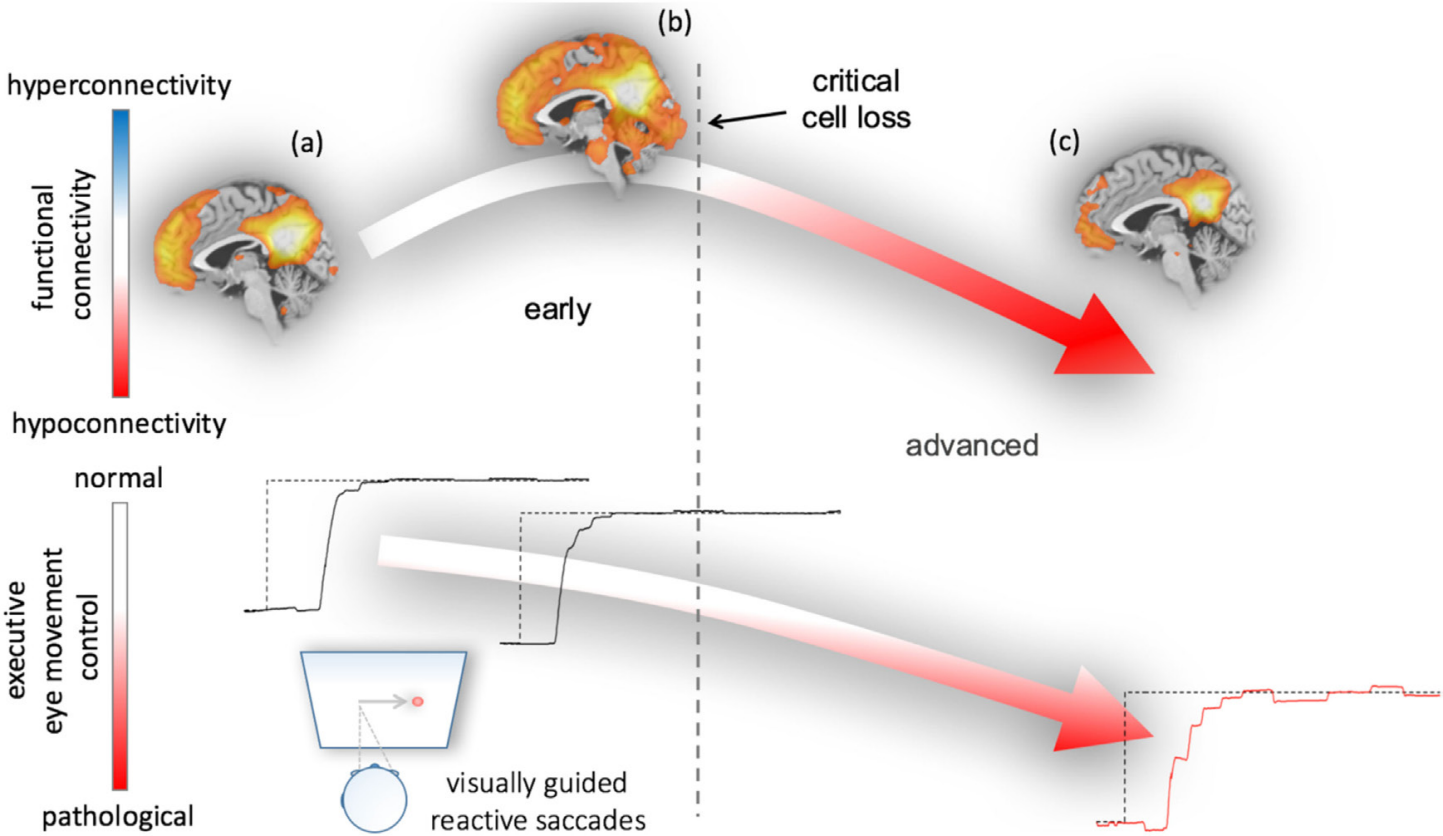
Hypothetical model of functional connectivity alterations in association with executive eye movement control in Parkinson’s disease (PD). The model results from correlations between functional connectivity data and eye movement impairment in early and advanced patients with PD (63). Neural damage due to the ongoing PD-associated pathological process from **(A)** healthy or premotor to **(B)** clinically manifest disease status paradoxically results in increased functionally connectivity early in the course of the disease upon a critical cell loss is reached. During this phase, executive oculomotor function gradually worsens as evidenced from visually guided reactive saccade performance (lower row)—remarkably, neuropsychological assessment in these patients revealed cognitively unimpaired “normal” performance (67). **(C)** In the final stages of PD, patients most patients met the criteria of PD-associated dementia and have developed a function disconnection syndrome (decreased functional connectivity) that is associated with a pattern of severely impaired eye movement control (right lower panel).

These findings raised the question whether functional connectivity between interconnected gray matter regions is correlated with oculomotor deficits. In a network-based rs-fMRI study in PD, a pronounced pattern of increased functional connectivity in cognitively unimpaired patients and a pattern of decreased functional connectivity in demented patients could be demonstrated (67). The pattern of abnormal functional connectivity is, in addition, related to abnormal oculomotor performance as revealed by a study of rs-fMRI and video-oculography in PD patients ranging from mild cognitive impairment to dementia (63). In particular, impaired executive oculomotor functions are correlated with a functional connectivity loss in the cognition-related default mode functional network. Taken together, these results allow for the development of a hypothetical model that links oculomotor performance and macro- and microstructural brain changes. Here, oculomotor performance markedly declined in the course of PD and functional connectivity appears to decrease after a critical cell loss has been reached; Figure 3 illustrates a hypothetical model of PD-associated alterations of functional connectivity together with executive eye movement control changes. The suggested course of functional connectivity is somewhat speculative, but many studies in the field of functional brain mapping try to establish a connection between neurodegeneration and adaptive mechanisms in relation to clinical phenotypes (68). We did not find any correlation between oculomotor parameters and volumetric, structural, and functional measures in ponto-cerebellar structures, midbrain or brainstem in PD—this may indicate that oculomotor deficits are not associated with disturbed ponto-cerebellar circuits or impaired oculomotor brainstem nuclei.

Previous studies supported the notion of a possible cerebellar involvement in PD (69) which has been recently strengthened by reports of α-synuclein aggregation in precerebellar structures (70). Connectivity studies in macaques (71) and DTI studies in humans (72) indicated that the cerebellum is part of a cerebello-cortico-basal ganglia network that is affected in PD. However, the role of this network and its alterations due to possibly impaired connectivity with respect to oculomotor function has not been systematically disentangled yet. Our oculomotor experience revealed a tendency toward a pattern of a “pontocerebellar type” of smooth pursuit disturbance in PD patients in an advanced disease state, most frequently accompanied by dementia. This observation leads to the speculative conclusion that the cerebellum, if ever, becomes involved later in the course of the disease as proposed by Braak and Del Tredici (60).

Given that, in PD, pathology progresses in different disease stages (60, 73) and eye movement performance worsen over time (30), various MRI techniques, i.e., volumetry, DTI, and task-based and rs-fMRI, allow to map cerebral correlates of oculomotor disturbances. This is specifically important to address the underlying disease-specific pathology both at a structural and functional level. According to the promotion of molecular pathology as suggested for PD (74), raises the question what has happened to the brain when oculomotor deficits manifest. Are these deficits driven by functional decline or structural damage or both? And which diseased brain regions are suspect to be associated with a particular pattern of eye movement disturbances? These questions cannot be fully addressed yet, but systematic studies investigating eye movement control and brain connectivity (75, 76) may indicate that network-based functional connectivity alterations are associated with worsened executive oculomotor function and that functional abnormalities may precede microstructural and macrostructural changes. Although the primary visual cortex in PD appears to be spared by the pathological process (60), a broad spectrum of visuo-oculomotor dysfunctions including color vision (77) and diplopia have been reported (78). Impaired color vision seems to be predictive to developing cognitive problems in PD (79) which could be most likely due to a network dysfunction involving vison-associated structures. Diplopia is frequently reported by PD patients and has been investigated in non-demented PD patients (78). From a clinical perspective, one might speculate that reporting diplopia in association with PD might be predictive for cognitive decline, but diplopia in association with the risk of developing cognitive dysfunctions remains to be investigated on a systematic basis.

Antiparkinsonian treatment including deep brain stimulation of the subthalamic nucleus was reported to improve oculomotor inhibition control and to facilitate saccade initiation (80), most likely due to compensatory mechanisms (81), whereas other groups reported no significantly improved oculomotor performance (62). Apparently, improvement of oculomotor performance due to antiparkinsonian treatment depends on the disease state, i.e., patients early in the course are more likely to improve eye movement performance (18). Future prospective studies are required to unravel the effect of treatment on eye movement performance in association with brain connectivity.

### Other Neurodegenerative Parkinsonism

Progressive supranuclear palsy and MSA are other parkinsonian syndromes (82–85) which comprise a characteristic spectrum of oculomotor dysfunctions (65). In contrast to PD patients who predominantly show oculomotor dysfunctions that are attributable to executive dysfunctions, PSP and MSA patients were shown to present predominantly “genuine” oculomotor dysfunctions (31). Impaired “genuine” oculomotor function comprised reduced peak eye velocity feature in PSP (3) which is used for diagnostic differentiation between PSP and both PD and MSA (30). Reduced peak eye velocity is the hallmark oculomotor feature in PSP patients (86) which is present at different levels from normal saccade velocity toward gaze palsy which has become increasingly relevant in the diagnostic guidelines for PSP (85). Earlier studies suggested that deficient generation of the motor command by midbrain burst neurons is most likely the cause of slowed vertical saccades in PSP (87).

**Figure 4:**
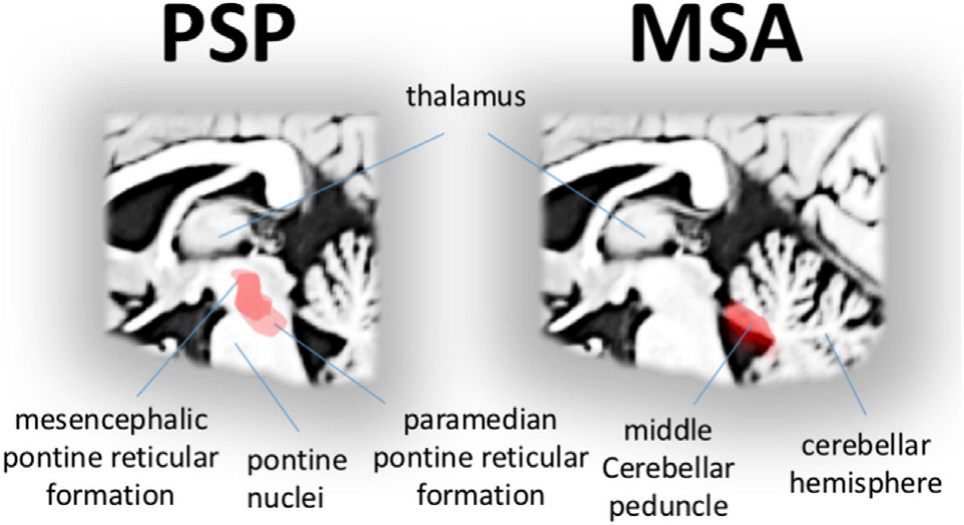
Disease-specific correlations of eye movement alterations in progressive supranuclear palsy (PSP) and multisystem atrophy (MSA). Specific correlations (red area) between microstructural impairment and gaze palsy in patients with PSP showing midbrain and brainstem regions typically associated with eye velocity (left). Specific correlations (red area) between microstructural impairment in ponto-cerebellar structures and the shape of saccadized smooth pursuit in patients with MSA (right).

Progressive supranuclear palsy is considered a neuropathologically defined disease presenting with a broad spectrum of clinical phenotypes besides the “classical” phenotype Richardson syndrome [PSP-RS (88)], including the Parkinsonian subtype (PSP-P), corticobasal syndrome subtype, and frontotemporal dementia subtypes (89) besides further variants which are of limited importance for oculomotor control. Slowed saccades in all subtypes of PSP are due to the paucity in burst generation at the excitatory burst (90). The PSP-RS and PSP-P subtypes show an almost identical oculomotor phenotype, hence, eye movement recordings do not allow to distinguish between PSP-PS and PSP-P (91). There are no systematic data for eye movement alterations associated with the other variants yet. It might be of note in that context that, in patients with frontal lobe degeneration, saccadic and smooth pursuit eye movements are impaired (92), and multimodal morphological studies revealed a link between atrophy in frontal brain regions and executive oculomotor performance (93). In the search of an imaging correlate of slowed saccades in PSP (including both PSP-RS and PSP-P patients), it could be demonstrated that the characteristic deficits in eye movement control were associated with regional macrostructural (41) and microstructural white matter alterations (94) (Figure 4, left). In particular, the hallmark oculomotor feature in PSP, a pathologically reduced peak eye velocity in both horizontal and (predominantly) vertical direction is associated with midbrain and brainstem pathology including the oculomotor nuclei responsible to “drive” the extra-ocular eye muscles. In addition, a recent “resting-state” fMRI study in both PSP-RS and PSP-P could also demonstrate correlations between midbrain functional connectivity and brainstem gaze centers (95). This finding is in agreement with the fact that on the one hand degeneration of neurons in the midbrain gaze centers in PSP-RS leads to progressive slowing of saccades (3) but that on the other hand slowed vertical saccades are not necessarily present in an early state of each subtype of PSP. In the tauopathy corticobasal degeneration as defined by Armstrong and coworkers in 2013 (96), it is mentioned that eye movement abnormalities may be present in about 60% of corticobasal degeneration cases, but data are sparse and heterogeneous, partly describing increased saccadic latency and abnormal antisaccade performance. However, studies on neuroimaging correlates are lacking. Taken together, this group of neurodegenerative disorders often overlaps clinically, and future studies have to investigate well-defined samples.

Multisystem atrophy can be distinguished in a cerebellar subtype (MSA-C) and a MSA-P. The oculomotor phenotype in MSA-C and MSA-P is almost identical (4) and does not allow to separate both subtypes—a finding that is supported by a recent bimodal MRI and VOG study that investigated functional connectivity and smooth pursuit performance in MSA-C and MSA-P patients (97).

Smooth pursuit eye movement is the ability to perfectly stabilizing the image of a continuously moving object onto the fovea (36). In MSA, smooth pursuit eye movement is disturbed throughout a reduced gain (i.e., eye velocity/target velocity <1), resulting in an offset between target and eye position, i.e., the target continuously slips away from the center of the fovea (18). This offset is almost instantaneously corrected by a “catch-up” saccade that centers the fovea back onto the target—an adaptive process that results in a staircase pattern without episodes of perfect smooth pursuit (4). The presence of catch-up saccades interrupting smooth pursuit is a common oculomotor feature in patients with ponto-cerebellar impairment like in MSA (4) and contributes to the differential diagnosis (18).

Lesion studies in animals that targeted vital elements of the smooth pursuit pathways including the cerebellar vermis and precerebellar nuclei, indicate that these structures are responsible for catch-up saccades (36). Consistent with this finding, severe macro- and microstructural damage in the bilateral middle cerebellar peduncles in MSA patients is correlated with smooth pursuit impairment (Figure 4, right). In particular, pontine volume loss is strongly related to the shape of saccadized smooth pursuit as demonstrated in a study of video-oculographically recorded eye movements and ABV-based volumetry (41). These findings are further strengthened by correlating performance measures of smooth pursuit with DTI-based measures of microstructural impairment. The degree of microstructural impairment in the middle cerebellar peduncle was strongly correlated with the shape of “catch-up” saccades during smooth pursuit in MSA (94). Moreover, abnormal functional connectivity within the ponto-cerebellar network is also strongly correlated with the shape of characteristically impaired smooth pursuit as revealed in rs-fMRI study in MSA patients compared to controls (97).

These findings, at a broader scope, may allow to generally speculate about brain structure and function in association with oculomotor phenotyping in parkinsonian syndromes. Disease-characteristic patterns of impaired oculomotor control gradually worsen over time and are apparently closely related with ongoing region-specific macrostructural and microstructural damage. The pattern of network-dependent functional connectivity alterations is more complex. As suggested, the pattern of functional connectivity increases and then gradually declines toward a disconnection syndrome. The development of functional connectivity in the course of the disease is well explained by the concept of adaptive changes (i.e., hyperconnectivity) that aims to compensate for ongoing cell loss in the sense of cortical network reorganization up to a point in time where a critical cell loss is reached (68, 98). From this point in time, compensation is no longer possible and the cognitive reserve is exhausted (99, 100). A limitation of the suggested model is the lack of information from longitudinal studies.

## A Perspective on Connectomics and Eye Movement Control

The brain is an efficient representation of a complex system (101, 102) which consists of spatially distributed and functionally specialized regions that continuously share information with each other (103). Graph-theoretical approaches for the analysis of both structural and functional networks enable to quantify properties of the brain’s functional system together with the underlying wiring (104). A network is defined in graph-theory as a set of nodes, i.e., anatomically segregated brain regions, and edges, i.e., a connectivity measure, between two nodes (105). Many measures of useful properties that characterize the network organization can be computed, including basic concepts, measures of segregation, integration, motifs, resilience, and other concepts such as “network small-worldness” (106). These measures are to be correlated with behavioral parameters including quantitative measures of eye movement control. For instance, the saccadic reaction times are prolonged in parkinsonian syndromes, but there is no report about any specific regions of the brain which are structurally or functionally correlated with reaction times (41, 63, 94). It appears that there is no single “region” associated with latency; rather, reaction time could be hypothesized to reflect network efficiency computable in a graph-based framework. Thus, modeling the brain connectivity as a graph, with nodes being segregated brain modules and edges being “region-to-region” connectivity strengths, opens a new avenue for investigating brain organization in association with the respective oculomotor phenotype in parkinsonian syndromes. Recent evidence strongly suggests that the anatomical connections determine whether they are vulnerable to degeneration in neurodegenerative parkinsonism (107). It remains to be investigated whether eye movements provide a window into the status of neurodegeneration (disease stage) or even allow to serve as a prognostic marker.

## Concluding Remarks

The oculomotor analysis of a patient using gaze-tracking technology might help clinicians to gain insights into the brain function and disease status. In addition, the reviewed studies pave the way toward the development of a standardized protocol for video-oculographic assessment in the differential diagnostic frame aiming at establishing a technical surrogate marker. In neurodegenerative parkinsonism, worse oculomotor performance in the disease-specific domain was shown to be associated with more severely impaired regional macro- and microstructure and altered regional functional connectivity in disease-specific brain structures. These findings increase our pathophysiological knowledge of the underlying parkinsonism-associated network pathology. Finally, brain mapping of impaired eye movement control as shown for parkinsonian syndromes should be investigated in a broader context of brain diseases in order to find out whether the demonstrated findings could be generalized to neurodegenerative diseases beyond parkinsonism.

## Author Contributions

MG and JK drafted the manuscript. H-PM revised the manuscript for intellectual content. All authors performed literature search, agreed to be accountable for the content of the work, and finally approved the manuscript.

## Conflict of Interest Statement

The authors declare that the research was conducted in the absence of any commercial or financial relationships that could be construed as a potential conflict of interest.

## References

[1] List CF Disturbances of eye movements as a neurologic problem. N Engl J Med (1956) 254:461–4. 10.1056/NEJM195603082541004 13297135

[2] Anderson TJ MacAskill MR. Eye movements in patients with neurodegenerative disorders. Nat Rev Neurol (2013) 9:74–85. 10.1038/nrneurol.2012.273 23338283

[3] Chen AL Riley DE King SA Joshi AC Serra A Liao K The disturbance of gaze in progressive supranuclear palsy: implications for pathogenesis. Front Neurol (2010) 1:147. 10.3389/fneur.2010.00147 21188269 PMC3008928

[4] Pinkhardt EH Kassubek J Süssmuth S Ludolph AC Becker W Jürgens R. Comparison of smooth pursuit eye movement deficits in multiple system atrophy and Parkinson’s disease. J Neurol (2009) 256:1438–46. 10.1007/s00415-009-5131-5 19363627

[5] Munoz DP Everling S Look away: the anti-saccade task and the voluntary control of eye movement. Nat Rev Neurosci (2004) 5:218–28. 10.1038/nrn1345 14976521

[6] Goldberg M Walker M The control of gaze. 5th ed In: James A Schwartz H Jessell T Siegelbaum S Kandel E, editors. Principles of Neural Science. New York, USA: McGraw-Hill (2013). p. 894–916.

[7] Krajbich I Rangel A. Multialternative drift-diffusion model predicts the relationship between visual fixations and choice in value-based decisions. Proc Natl Acad Sci U S A (2011) 108:13852–7. 10.1073/pnas.1101328108 21808009 PMC3158210

[8] Pärnamets P Johansson P Hall L Balkenius C Spivey MJ Richardson DC. Biasing moral decisions by exploiting the dynamics of eye gaze. Proc Natl Acad Sci U S A (2015) 112:4170–5. 10.1073/pnas.1415250112 25775604 PMC4386374

[9] Krajbich I Armel C Rangel A. Visual fixations and the computation and comparison of value in simple choice. Nat Neurosci (2010) 13:1292–8. 10.1038/nn.2635 20835253

[10] Leigh RJ Zee DS The Neurology of Eye Movements. 5th ed Oxford; New York: Oxford University Press (2015).

[11] Ettinger U Antonova E Crawford TJ Mitterschiffthaler MT Goswani S Sharma T Structural neural correlates of prosaccade and antisaccade eye movements in healthy humans. Neuroimage (2005) 24:487–94. 10.1016/j.neuroimage.2004.08.019 15627590

[12] Sharika KM Neggers SFW Gutteling TP Van der Stigchel S Dijkerman HC Murthy A. Proactive control of sequential saccades in the human supplementary eye field. Proc Natl Acad Sci U S A (2013) 110:E1311–20. 10.1073/pnas.1210492110 23493559 PMC3619306

[13] Jamadar SD Fielding J Egan GF. Quantitative meta-analysis of fMRI and PET studies reveals consistent activation in fronto-striatal-parietal regions and cerebellum during antisaccades and prosaccades. Front Psychol (2013) 4:749. 10.3389/fpsyg.2013.00749 24137150 PMC3797465

[14] Ramot M Wilf M Goldberg H Weiss T Deouell LY Malach R. Coupling between spontaneous (resting state) fMRI fluctuations and human oculo-motor activity. Neuroimage (2011) 58:213–25. 10.1016/j.neuroimage.2011.06.015 21703354

[15] Pierrot-Deseilligny C Milea D Müri RM. Eye movement control by the cerebral cortex. Curr Opin Neurol (2004) 17:17–25. 10.1097/00019052-200402000-00005 15090873

[16] Yerram S Glazman S Bodis-Wollner I. Cortical control of saccades in Parkinson disease and essential tremor. J Neural Transm (Vienna) (2013) 120:145–56. 10.1007/s00702-012-0870-3 22926662

[17] Pievani M Filippini N van den Heuvel MP Cappa SF Frisoni GB Brain connectivity in neurodegenerative diseases – from phenotype to proteinopathy. Nat Rev Neurol (2014) 10:620–33. 10.1038/nrneurol.2014.178 25287597

[18] Gorges M Pinkhardt EH Kassubek J. Alterations of eye movement control in neurodegenerative movement disorders. J Ophthalmol (2014) 2014:658243. 10.1155/2014/658243 24955249 PMC4052189

[19] Gorges M Müller H-P Ludolph AC Rasche V Kassubek J. Intrinsic functional connectivity networks in healthy elderly subjects: a multiparametric approach with structural connectivity analysis. Biomed Res Int (2014) 2014:947252. 10.1155/2014/947252 24971361 PMC4058120

[20] Schreiber K Haslwanter T. Improving calibration of 3-D video oculography systems. IEEE Trans Biomed Eng (2004) 51:676–9. 10.1109/TBME.2003.821025 15072222

[21] Zwergal A Brandt T Magnusson M Kennard C DIZZYNET – a European network initiative for vertigo and balance research: visions and aims. J Neurol (2016) 263(Suppl 1):S2–9. 10.1007/s00415-015-7912-3 27083879 PMC4833796

[22] Pinkhardt EH Kassubek J. Ocular motor abnormalities in parkinsonian syndromes. Parkinsonism Relat Disord (2011) 17:223–30. 10.1016/j.parkreldis.2010.08.004 20801069

[23] Keller J Gorges M Aho-Özhan HEA Uttner I Schneider E Kassubek J Eye-tracking control to assess cognitive functions in patients with amyotrophic lateral sclerosis. J Vis Exp (2016). 10.3791/54634 27768047 PMC5092192

[24] Schneider E Villgrattner T Vockeroth J Bartl K Kohlbecher S Bardins S EyeSeeCam: an eye movement-driven head camera for the examination of natural visual exploration. Ann N Y Acad Sci (2009) 1164:461–7. 10.1111/j.1749-6632.2009.03858.x 19645949

[25] Helmchen C Pohlmann J Trillenberg P Lencer R Graf J Sprenger A. Role of anticipation and prediction in smooth pursuit eye movement control in Parkinson’s disease. Mov Disord (2012) 27:1012–8. 10.1002/mds.25042 22693071

[26] Gorges M Müller H-P Lulé D Ludolph AC Pinkhardt EH Kassubek J. Functional connectivity within the default mode network is associated with saccadic accuracy in Parkinson’s disease: a resting-state fMRI and videooculographic study. Brain Connect (2013) 3:265–72. 10.1089/brain.2013.0146 23627641

[27] Becker W The neurobiology of saccadic eye movements. Metrics. Rev Oculomot Res (1989) 3:13–67. 2486323

[28] Hallett PE Primary and secondary saccades to goals defined by instructions. Vision Res (1978) 18:1279–96. 10.1016/0042-6989(78)90218-3 726270

[29] Becker W Do correction saccades depend exclusively on retinal feedback? A note on the possible role of non-retinal feedback. Vision Res (1976) 16:425–7. 10.1016/0042-6989(76)90209-1 941420

[30] MacAskill MR Anderson TJ. Eye movements in neurodegenerative diseases. Curr Opin Neurol (2016) 29:61–8. 10.1097/WCO.0000000000000274 26641817

[31] Pinkhardt EH Issa H Gorges M Jürgens R Lulé D Heimrath J Do eye movement impairments in patients with small vessel cerebrovascular disease depend on lesion load or on cognitive deficits? A video-oculographic and MRI study. J Neurol (2014) 261:791–803. 10.1007/s00415-014-7275-1 24535136

[32] Kassubek J Danek A Del Tredici-Braak K Greenlee MW Pinkhardt EH [The eye as a window to the pathophysiology in Parkinson’s syndromes]. Nervenarzt (2013) 84:909–17. 10.1007/s00115-013-3754-3 23760595

[33] Sparks DL The brainstem control of saccadic eye movements. Nat Rev Neurosci (2002) 3:952–64. 10.1038/nrn986 12461552

[34] Hikosaka O Takikawa Y Kawagoe R. Role of the basal ganglia in the control of purposive saccadic eye movements. Physiol Rev (2000) 80:953–78. 10.1152/physrev.2000.80.3.953 10893428

[35] Corbetta M Akbudak E Conturo TE Snyder AZ Ollinger JM Drury HA A common network of functional areas for attention and eye movements. Neuron (1998) 21:761–73. 10.1016/S0896-6273(00)80593-0 9808463

[36] Fukushima K Fukushima J Warabi T Barnes GR. Cognitive processes involved in smooth pursuit eye movements: behavioral evidence, neural substrate and clinical correlation. Front Syst Neurosci (2013) 7:4. 10.3389/fnsys.2013.00004 23515488 PMC3601599

[37] Nenadic I Maitra R Langbein K Dietzek M Lorenz C Smesny S Brain structure in schizophrenia vs. psychotic bipolar I disorder: a VBM study. Schizophr Res (2015) 165:212–9. 10.1016/j.schres.2015.04.007 25935815

[38] Walton CC O’Callaghan C Hall JM Gilat M Mowszowski L Naismith SL Antisaccade errors reveal cognitive control deficits in Parkinson’s disease with freezing of gait. J Neurol (2015) 262:2745–54. 10.1007/s00415-015-7910-5 26464101

[39] Huppertz H-J Kröll-Seger J Klöppel S Ganz RE Kassubek J. Intra- and interscanner variability of automated voxel-based volumetry based on a 3D probabilistic atlas of human cerebral structures. Neuroimage (2010) 49:2216–24. 10.1016/j.neuroimage.2009.10.066 19878722

[40] Huppertz H-J Möller L Südmeyer M Hilker R Hattingen E Egger K Differentiation of neurodegenerative parkinsonian syndromes by volumetric magnetic resonance imaging analysis and support vector machine classification. Mov Disord (2016) 31:1506–17. 10.1002/mds.26715 27452874

[41] Vintonyak O Gorges M Müller H-P Pinkhardt EH Ludolph AC Huppertz H-J Patterns of eye movement impairment correlate with regional brain atrophy in neurodegenerative parkinsonism. Neurodegener Dis (2017) 17:117–26. 10.1159/000454880 28268209

[42] Mori S Zhang J. Principles of diffusion tensor imaging and its applications to basic neuroscience research. Neuron (2006) 51:527–39. 10.1016/j.neuron.2006.08.012 16950152

[43] Müller H-P Kassubek J Diffusion tensor magnetic resonance imaging in the analysis of neurodegenerative diseases. J Vis Exp (2013). 10.3791/50427 PMC3846464 23928996

[44] Cochrane CJ Ebmeier KP. Diffusion tensor imaging in parkinsonian syndromes: a systematic review and meta-analysis. Neurology (2013) 80:857–64. 10.1212/WNL.0b013e318284070c 23439701 PMC3598454

[45] Bosch SE Neggers SFW Van der Stigchel S. The role of the frontal eye fields in oculomotor competition: image-guided TMS enhances contralateral target selection. Cereb Cortex (2013) 23:824–32. 10.1093/cercor/bhs075 22455840

[46] Haynes J-D. A primer on pattern-based approaches to fMRI: principles, pitfalls, and perspectives. Neuron (2015) 87:257–70. 10.1016/j.neuron.2015.05.025 26182413

[47] Schmid A Rees G Frith C Barnes G. An fMRI study of anticipation and learning of smooth pursuit eye movements in humans. Neuroreport (2001) 12:1409–14. 10.1097/00001756-200105250-00023 11388420

[48] Tanabe J Tregellas J Miller D Ross RG Freedman R. Brain activation during smooth-pursuit eye movements. Neuroimage (2002) 17:1315–24. 10.1006/nimg.2002.1263 12414271

[49] Sweeney JA Luna B Keedy SK McDowell JE Clementz BA. fMRI studies of eye movement control: investigating the interaction of cognitive and sensorimotor brain systems. Neuroimage (2007) 36(Suppl 2):T54–60. 10.1016/j.neuroimage.2007.03.018 17499170 PMC2692203

[50] Lemos J Pereira D Almendra L Rebelo D Patrício M Castelhano J Distinct functional properties of the vertical and horizontal saccadic network in health and Parkinson’s disease: an eye-tracking and fMRI study. Brain Res (2016) 1648:469–84. 10.1016/j.brainres.2016.07.037 27545665

[51] Lemos J Pereira D Almendra L Rebelo D Patrício M Castelhano J Cortical control of vertical and horizontal saccades in progressive supranuclear palsy: an exploratory fMRI study. J Neurol Sci (2017) 373:157–66. 10.1016/j.jns.2016.12.049 28131178

[52] Baggio H-C Segura B Sala-Llonch R Marti M-J Valldeoriola F Compta Y Cognitive impairment and resting-state network connectivity in Parkinson’s disease. Hum Brain Mapp (2015) 36:199–212. 10.1002/hbm.22622 25164875 PMC6869118

[53] Biswal B Yetkin FZ Haughton VM Hyde JS. Functional connectivity in the motor cortex of resting human brain using echo-planar MRI. Magn Reson Med (1995) 34:537–41. 10.1002/mrm.1910340409 8524021

[54] Raichle ME. The restless brain: how intrinsic activity organizes brain function. Philos Trans R Soc Lond B Biol Sci (2015) 370. 10.1098/rstb.2014.0172 25823869 PMC4387513

[55] Park H-J Friston K. Structural and functional brain networks: from connections to cognition. Science (2013) 342:1238411. 10.1126/science.1238411 24179229

[56] Power JD Schlaggar BL Petersen SE. Studying brain organization via spontaneous fMRI signal. Neuron (2014) 84:681–96. 10.1016/j.neuron.2014.09.007 25459408 PMC4254503

[57] Smith SM Fox PT Miller KL Glahn DC Fox PM Mackay CE Correspondence of the brain’s functional architecture during activation and rest. Proc Natl Acad Sci U S A (2009) 106:13040–5. 10.1073/pnas.0905267106 19620724 PMC2722273

[58] Biswal BB. Resting state fMRI: a personal history. Neuroimage (2012) 62:938–44. 10.1016/j.neuroimage.2012.01.090 22326802 PMC12911935

[59] Laird AR Fox PM Eickhoff SB Turner JA Ray KL McKay DR Behavioral interpretations of intrinsic connectivity networks. J Cogn Neurosci (2011) 23:4022–37. 10.1162/jocn_a_00077 21671731 PMC3690655

[60] Braak H Del Tredici K. Neuroanatomy and pathology of sporadic Parkinson’s disease. Adv Anat Embryol Cell Biol (2009) 201:1–119. 19230552

[61] Braak H Del Tredici K. Potential pathways of abnormal tau and α-synuclein dissemination in sporadic Alzheimer’s and Parkinson’s diseases. Cold Spring Harb Perspect Biol (2016) 8. 10.1101/cshperspect.a023630 27580631 PMC5088528

[62] Pinkhardt EH Jürgens R Lulé D Heimrath J Ludolph AC Becker W Eye movement impairments in Parkinson’s disease: possible role of extradopaminergic mechanisms. BMC Neurol (2012) 12:5. 10.1186/1471-2377-12-5 22375860 PMC3306761

[63] Gorges M Müller H-P Lulé D LANDSCAPE Consortium Pinkhardt EH Ludolph AC The association between alterations of eye movement control and cerebral intrinsic functional connectivity in Parkinson’s disease. Brain Imaging Behav (2016) 10:79–91. 10.1007/s11682-015-9367-7 25749936

[64] Mosimann UP Müri RM Burn DJ Felblinger J O’Brien JT McKeith IG. Saccadic eye movement changes in Parkinson’s disease dementia and dementia with Lewy bodies. Brain (2005) 128:1267–76. 10.1093/brain/awh484 15774501

[65] Kassubek J Pinkhardt EH Neuro-ophthalmological alterations in patients with movement disorders. In: Galvez-Jimenez N Tuite P, editors. Uncommon Causes of Movement Disorders (2011). Cambridge: Cambridge University Press p. 306–15.

[66] Baggio H-C Sala-Llonch R Segura B Marti M-J Valldeoriola F Compta Y Functional brain networks and cognitive deficits in Parkinson’s disease: functional network analysis in PD. Hum Brain Mapp (2014) 35:4620–34. 10.1002/hbm.22499 24639411 PMC6869398

[67] Gorges M Müller H-P Lulé D LANDSCAPE Consortium Pinkhardt EH Ludolph AC To rise and to fall: functional connectivity in cognitively normal and cognitively impaired patients with Parkinson’s disease. Neurobiol Aging (2015) 36:1727–35. 10.1016/j.neurobiolaging.2014.12.026 25623332

[68] Hillary FG Grafman JH. Injured brains and adaptive networks: the benefits and costs of hyperconnectivity. Trends Cogn Sci (Regul Ed) (2017) 21:385–401. 10.1016/j.tics.2017.03.003 28372878 PMC6664441

[69] Wu T Hallett M. The cerebellum in Parkinson’s disease. Brain (2013) 136:696–709. 10.1093/brain/aws360 23404337 PMC7273201

[70] Seidel K Bouzrou M Heidemann N Krüger R Schöls L den Dunnen WFA Involvement of the cerebellum in Parkinson disease and dementia with Lewy bodies. Ann Neurol (2017) 81:898–903. 10.1002/ana.24937 28439961

[71] Hoshi E Tremblay L Féger J Carras PL Strick PL. The cerebellum communicates with the basal ganglia. Nat Neurosci (2005) 8:1491–3. 10.1038/nn1544 16205719

[72] Milardi D Arrigo A Anastasi G Cacciola A Marino S Mormina E Extensive direct subcortical cerebellum-basal ganglia connections in human brain as revealed by constrained spherical deconvolution tractography. Front Neuroanat (2016) 10:29. 10.3389/fnana.2016.00029 27047348 PMC4796021

[73] Braak H Rüb U Gai WP Del Tredici K. Idiopathic Parkinson’s disease: possible routes by which vulnerable neuronal types may be subject to neuroinvasion by an unknown pathogen. J Neural Transm (Vienna) (2003) 110:517–36. 10.1007/s00702-002-0808-2 12721813

[74] Schapira AHV Olanow CW Greenamyre JT Bezard E. Slowing of neurodegeneration in Parkinson’s disease and Huntington’s disease: future therapeutic perspectives. Lancet (2014) 384:545–55. 10.1016/S0140-6736(14)61010-2 24954676

[75] Gorges M Rosskopf J Müller H-P Lindemann K Hornyak M Kassubek J. Patterns of increased intrinsic functional connectivity in patients with restless legs syndrome are associated with attentional control of sensory inputs. Neurosci Lett (2016) 617:264–9. 10.1016/j.neulet.2016.02.043 26921454

[76] Gorges M Müller H-P Mayer IMS Grupe GS Kammer T Grön G Intact sensory-motor network structure and function in far from onset premanifest Huntington’s disease. Sci Rep (2017) 7:43841. 10.1038/srep43841 28266655 PMC5339687

[77] Armstrong RA. Visual dysfunction in Parkinson’s disease. Int Rev Neurobiol (2017) 134:921–46. 10.1016/bs.irn.2017.04.007 28805589

[78] Schindlbeck KA Schönfeld S Naumann W Friedrich DJ Maier A Rewitzer C Characterization of diplopia in non-demented patients with Parkinson’s disease. Parkinsonism Relat Disord (2017) 45:1–6. 10.1016/j.parkreldis.2017.09.024 28993094

[79] Anang JBM Gagnon J-F Bertrand J-A Romenets SR Latreille V Panisset M Predictors of dementia in Parkinson disease: a prospective cohort study. Neurology (2014) 83:1253–60. 10.1212/WNL.0000000000000842 25171928 PMC4180482

[80] Yugeta A Terao Y Fukuda H Hikosaka O Yokochi F Okiyama R Effects of STN stimulation on the initiation and inhibition of saccade in Parkinson disease. Neurology (2010) 74:743–8. 10.1212/WNL.0b013e3181d31e0b 20194913

[81] Nilsson MH Patel M Rehncrona S Magnusson M Fransson P-A. Subthalamic deep brain stimulation improves smooth pursuit and saccade performance in patients with Parkinson’s disease. J Neuroeng Rehabil (2013) 10:33. 10.1186/1743-0003-10-33 23551890 PMC3621588

[82] Stefanova N Bücke P Duerr S Wenning GK. Multiple system atrophy: an update. Lancet Neurol (2009) 8:1172–8. 10.1016/S1474-4422(09)70288-1 19909915

[83] Ubhi K Low P Masliah E. Multiple system atrophy: a clinical and neuropathological perspective. Trends Neurosci (2011) 34:581–90. 10.1016/j.tins.2011.08.003 21962754 PMC3200496

[84] Boxer AL Yu J-T Golbe LI Litvan I Lang AE Höglinger GU. Advances in progressive supranuclear palsy: new diagnostic criteria, biomarkers, and therapeutic approaches. Lancet Neurol (2017) 16:552–63. 10.1016/S1474-4422(17)30157-6 28653647 PMC5802400

[85] Höglinger GU Respondek G Stamelou M Kurz C Josephs KA Lang AE Clinical diagnosis of progressive supranuclear palsy: the movement disorder society criteria. Mov Disord (2017) 32:853–64. 10.1002/mds.26987 28467028 PMC5516529

[86] Marx S Respondek G Stamelou M Dowiasch S Stoll J Bremmer F Validation of mobile eye-tracking as novel and efficient means for differentiating progressive supranuclear palsy from Parkinson’s disease. Front Behav Neurosci (2012) 6:88. 10.3389/fnbeh.2012.00088 23248593 PMC3521127

[87] Bhidayasiri R Riley DE Somers JT Lerner AJ Büttner-Ennever JA Leigh RJ. Pathophysiology of slow vertical saccades in progressive supranuclear palsy. Neurology (2001) 57:2070–7. 10.1212/WNL.57.11.2070 11739828

[88] Respondek G Kurz C Arzberger T Compta Y Englund E Ferguson LW Which ante mortem clinical features predict progressive supranuclear palsy pathology? Mov Disord (2017) 32:995–1005. 10.1002/mds.27034 28500752 PMC5543934

[89] Respondek G Stamelou M Kurz C Ferguson LW Rajput A Chiu WZ The phenotypic spectrum of progressive supranuclear palsy: a retrospective multicenter study of 100 definite cases. Mov Disord (2014) 29:1758–66. 10.1002/mds.26054 25370486

[90] Shaikh AG Factor SA Juncos J. Saccades in progressive supranuclear palsy – maladapted, irregular, curved, and slow. Mov Disord Clin Pract (2017) 4:671–81. 10.1002/mdc3.12491 29333474 PMC5764187

[91] Pinkhardt EH Jürgens R Becker W Valdarno F Ludolph AC Kassubek J Differential diagnostic value of eye movement recording in PSP-parkinsonism, Richardson’s syndrome, and idiopathic Parkinson’s disease. J Neurol (2008) 255:1916–25. 10.1007/s00415-009-0027-y 19224319

[92] Boxer AL Garbutt S Seeley WW Jafari A Heuer HW Mirsky J Saccade abnormalities in autopsy-confirmed frontotemporal lobar degeneration and Alzheimer disease. Arch Neurol (2012) 69:509–17. 10.1001/archneurol.2011.1021 22491196 PMC3423186

[93] Boxer AL Garbutt S Rankin KP Hellmuth J Neuhaus J Miller BL Medial versus lateral frontal lobe contributions to voluntary saccade control as revealed by the study of patients with frontal lobe degeneration. J Neurosci (2006) 26:6354–63. 10.1523/JNEUROSCI.0549-06.2006 16763044 PMC2551317

[94] Gorges M Maier MN Rosskopf J Vintonyak O Pinkhardt EH Ludolph AC Regional microstructural damage and patterns of eye movement impairment: a DTI and video-oculography study in neurodegenerative parkinsonian syndromes. J Neurol (2017) 264:1919–28. 10.1007/s00415-017-8579-8 28762086

[95] Rosskopf J Gorges M Müller H-P Lulé D Uttner I Ludolph AC Intrinsic functional connectivity alterations in progressive supranuclear palsy: differential effects in frontal cortex, motor, and midbrain networks. Mov Disord (2017) 32:1006–15. 10.1002/mds.27039 28544256

[96] Armstrong MJ Litvan I Lang AE Bak TH Bhatia KP Borroni B Criteria for the diagnosis of corticobasal degeneration. Neurology (2013) 80:496–503. 10.1212/WNL.0b013e31827f0fd1 23359374 PMC3590050

[97] Rosskopf J Gorges M Müller H-P Pinkhardt EH Ludolph AC Kassubek J. Hyperconnective and hypoconnective cortical and subcortical functional networks in multiple system atrophy. Parkinsonism Relat Disord (2018) 49:75–80. 10.1016/j.parkreldis.2018.01.012 29352721

[98] Hillary FG Roman CA Venkatesan U Rajtmajer SM Bajo R Castellanos ND. Hyperconnectivity is a fundamental response to neurological disruption. Neuropsychology (2015) 29:59–75. 10.1037/neu0000110 24933491

[99] Stern Y Cognitive reserve and Alzheimer disease. Alzheimer Dis Assoc Disord (2006) 20:112–7. 10.1097/00002093-200607001-00010 16772747

[100] Stern Y. Cognitive reserve in ageing and Alzheimer’s disease. Lancet Neurol (2012) 11:1006–12. 10.1016/S1474-4422(12)70191-6 23079557 PMC3507991

[101] Bullmore E Sporns O. The economy of brain network organization. Nat Rev Neurosci (2012) 13:336–49. 10.1038/nrn3214 22498897

[102] Petri G Expert P Turkheimer F Carhart-Harris R Nutt D Hellyer PJ Homological scaffolds of brain functional networks. J R Soc Interface (2014) 11:20140873. 10.1098/rsif.2014.0873 25401177 PMC4223908

[103] van den Heuvel MP Hulshoff Pol HE. Exploring the brain network: a review on resting-state fMRI functional connectivity. Eur Neuropsychopharmacol (2010) 20:519–34. 10.1016/j.euroneuro.2010.03.008 20471808

[104] Bullmore E Sporns O. Complex brain networks: graph theoretical analysis of structural and functional systems. Nat Rev Neurosci (2009) 10:186–98. 10.1038/nrn2575 19190637

[105] van den Heuvel MP Sporns O. Rich-club organization of the human connectome. J Neurosci (2011) 31:15775–86. 10.1523/JNEUROSCI.3539-11.2011 22049421 PMC6623027

[106] Rubinov M Sporns O. Complex network measures of brain connectivity: uses and interpretations. Neuroimage (2010) 52:1059–69. 10.1016/j.neuroimage.2009.10.003 19819337

[107] Raj A Kuceyeski A Weiner M A network diffusion model of disease progression in dementia. Neuron (2012) 73:1204–15. 10.1016/j.neuron.2011.12.040 22445347 PMC3623298

